# Changes in Unemployment Affect Sickness Absence and Disability Retirement Rates: A Municipality-Level Panel Study

**DOI:** 10.3390/ijerph18126359

**Published:** 2021-06-11

**Authors:** Jenni Blomgren, Mikko Laaksonen, Riku Perhoniemi

**Affiliations:** 1 The Social Insurance Institution of Finland, Nordenskiöldinkatu 12, 00250 Helsinki, Finland; riku.perhoniemi@kela.fi; 2 Finnish Centre for Pensions, Tukkutorinkuja 5, 00580 Helsinki, Finland; mikko.laaksonen@etk.fi

**Keywords:** unemployment, sickness absence, disability retirement, disability benefits, municipalities, Finland

## Abstract

To enhance understanding of the interplay between unemployment and sickness absence and disability retirement, the aim of this study was to examine how changes in area-level unemployment rates are associated with changes in sickness absence and disability retirement rates in a longitudinal setting. Municipality-level time-series data were collected on unemployment, sickness absence, disability retirement and covariates from databases for Finnish municipalities for years 2003–2017 (*n* = 4425 municipality–year observations). Fixed effects panel regression models were used to analyse how changes in unemployment rates predict changes in sickness absence and disability retirement rates when comparing consecutive years. The results showed that when examining yearly cross-sections, a higher level of unemployment in the municipality was associated with higher sickness absence and disability retirement rates. However, longitudinal assessment of consecutive years with panel regression models showed that a one percentage point increase in the municipality-level unemployment rate was associated with a decrease both in the sickness absence rate (−1.3%, *p* < 0.001) and in the disability retirement rate (−2.1%, *p* = 0.011), adjusted for simultaneous changes in demographic and socio-economic covariates, morbidity and economic situation of the municipality. The results indicate that unemployment and disability benefits partly act as substitutes for each other. Unemployment and disability rates should be assessed together to reach a more complete understanding of the level of non-employment overall and in different areas.

## 1. Introduction

In the ageing European countries, prolonging working lives has become an important goal [1]. Unemployment and work-related disability, which may be manifested in sickness absence (short-term disability) or disability retirement (long-term or permanent disability), are the two main reasons that shorten working careers. Unemployment and disability risks are also interrelated. Several studies have shown that unemployment is positively associated with an individual’s risks of later sickness absence and disability retirement [2,3,4,5,6,7,8,9,10,11]. The literature has attributed this positive association to causation and selection that are at work simultaneously: unemployment may have adverse effects on health (=causation), but on the other hand, health problems and weak work ability also increase the risk of unemployment (=selection) [12,13,14,15]. Furthermore, a high area-level unemployment rate has also been shown to increase an individual’s sickness absence and disability pensioning risks [8,9,10,11,12]. Accordingly, studies using aggregate level data have shown positive area level cross-sectional correlations: higher area-level unemployment rates are associated with higher area-level disability rates [16,17,18].

However, macro-level trends tend to show a negative (a pro-cyclical) longitudinal association between the levels of unemployment and sickness absence: with a decreasing unemployment rate in a population, the sickness absence rate tends to increase, and correspondingly, with an increasing unemployment rate, sickness absence decreases [19,20,21,22,23]. The literature has suggested two main mechanisms for this association. The first is the differing composition of the workforce in terms of health depending on the economic cycle: during economic booms, those with work-ability problems are more likely to gain employment but they may also be prone to be absent due to sickness while employed. In contrast, during economic downturns, those with poorer work ability are more likely to lose their jobs or not gain employment in the first place. Their income is guaranteed through unemployment benefits, which then decreases the average take-up rate of sickness absence and disability retirement benefits [19,23,24,25,26]. The second suggested mechanism is the so-called moral hazard effect: during economic downturns, employed persons are afraid of losing their jobs and thus tend to avoid being absent, whereas the opposite is true during economic booms [19,23,24,25,27]. However, not all studies have found a pro-cyclical pattern: a Swedish study found no consistent pattern between macro-level unemployment and sickness absence rates [28]. An Icelandic study, utilizing country-level macro data, found that contrary to the pro-cyclical pattern, an increasing unemployment rate resulted in increasing, not decreasing, disability pension rolls. This suggests that unemployed persons with health problems may seek a more permanent solution for their long-term income source when employment prospects seem scarce [29]. This result seems to diverge from those received on the macro-level associations between unemployment and sickness absence. However, research on the macro-level associations between unemployment and disability retirement is lacking, especially utilizing data on different geographic levels, and more studies are needed to better understand these linkages.

This study aimed to increase understanding of the interplay between unemployment and use of sickness absence and disability retirement benefits by examining these associations utilizing panel data of Finnish municipalities. Previous studies analyzing area-level cross-sectional data have found a positive correlation between unemployment rates and disability rates [16,17,18], echoing results of individual-level studies. However, what about area-level dynamics in time—can they shed more light into the above-mentioned overall contradiction between individual-level and macro-level studies? We are not aware of previous studies utilizing area-level longitudinal data to explicitly examine these associations. Using municipality-level time-series data covering 15 years, the aim of the study was to examine how changes in the municipalities’ unemployment rates are associated with (1) changes in the sickness absence rates and (2) changes in the disability retirement rates in a longitudinal setting, adjusting for several municipality-level covariates.

## 2. Materials and Methods

### 2.1. Observations

Yearly area-level data on unemployment, sickness absence and disability retirement as well as covariates were collected on all municipalities of mainland Finland (*n* = 295) across 15 years (2003–2017), resulting in 4425 municipality–year observations in a cross-section time-series panel. Data were collected from databases maintained by Statistics Finland, Finnish Institute for Health and Welfare, the Social Insurance Institution of Finland and Finnish Centre for Pensions. The variables and their sources are presented in Table 1 and in Table A1 (Appendix A). As all data were collected from statistical databases at the aggregate level, no ethical review was required for the study.

Municipalities form the core of local administration in Finland. They are responsible for organizing statutory services for their inhabitants, such as health care and social services, day care, basic education, as well as cultural, transport and technical services. There have been some mergers of municipalities during years 2003–2017 but all data collected for the study were harmonized to follow the municipality structure during data collection in 2019.

### 2.2. Dependent Variables

Focusing on the two major indicators of work disability, the analyses were conducted separately on the yearly long-term sickness absence rate, i.e., the proportion (%) of persons aged 18–64 receiving sickness allowance during the year, and yearly disability retirement rate, i.e., the proportion (%) of persons aged 18–64 transferring to disability pension during the year. Both indicators were calculated relative to the non-retired population in the same age bracket.

Sickness allowance, paid by the Social Insurance Institution of Finland, compensates for income losses due to medically certified sickness absence after a waiting period of 10 working days, which are normally covered by the employer through sick pay. All persons aged 16–67 not on pensions are covered by the sickness allowance scheme. Sickness allowance can normally be paid for a maximum period of approximately one year. If incapacity to work lasts longer, disability pension may be granted after careful consideration by the pension insurers. Our measure of sickness absence rate includes persons who received sickness allowance for sickness absences longer than 10 working days at least once during the observation year.

Disability pension may be paid from the earnings-related scheme and/or from the national pension scheme that guarantees a minimum level of pension income to those with low or no earnings-related pensions. Our measure of disability retirement rate includes persons who transferred to disability retirement during the year and had no previous retirement events during two preceding years. Disability pensions usually start as fixed-term pensions, but those who are awarded a pension typically stay on disability pensions for the years to come and only rarely return to work [30].

The average annual sickness absence rate in the data set was 12% across all municipalities and years, and the average disability retirement rate 1%, respectively (Table 1).

### 2.3. Independent Variables

The main independent variable of interest was the yearly unemployment rate (%) of each municipality’s labour force. The average unemployment rate across all years and municipalities was 11% (Table 1).

Covariates included municipalities’ characteristics such as population and population density (used as logged variables), age and gender structures, socio-economic conditions, morbidity, economic situation as well as industry structure, as these were deemed important covariates in light of previous studies [11,16,25]. Most variables were available for all country-year observation points (*n* = 4425). However, variables describing the industry structure, i.e., proportions of those working on different fields of the economy, were comparably available only for years 2007–2017 (*n* = 3245).

**Figure A1:**
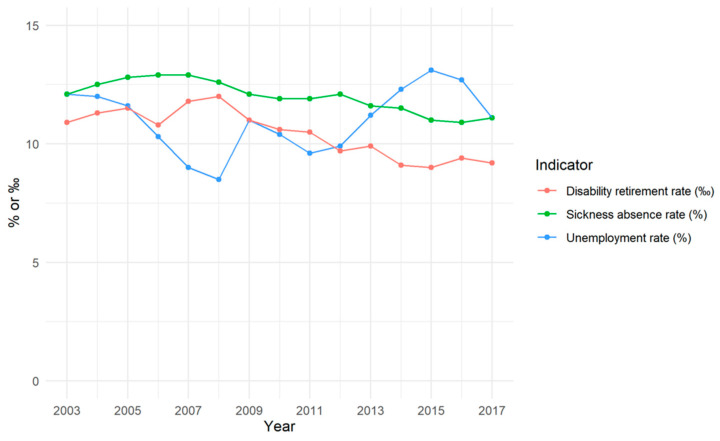
Average unemployment rate (%), sickness absence rate (%) and disability retirement rate (‰) in 2003–2017 calculated from unweighted municipality-level yearly observations.

Table 1 shows descriptive statistics for all variables in the data set pooled across all municipalities and years. Furthermore, Figure A1 (Appendix A) shows the overall trends of unemployment, sickness absence and disability retirement rates in 2003–2017, calculated as yearly averages of non-weighted municipality-level observations. Hints of a pro-cyclical pattern between unemployment and disability measures can be seen when looking at the average rates across years.

### 2.4. Statistical Modelling Strategy

Between-municipality correlations of unemployment and disability benefit rates were first examined to describe cross-sectional patterns. The data were further analysed with fixed-effects linear regression models, utilizing the panel structure of the data set [31]. Fixed-effects models automatically control for the fixed (time-constant) observed and unobserved characteristics of municipalities. Such time-constant factors include, for example, the physical location of the municipality. In fixed-effects models, only within-municipality variation is utilized; thus, the results reveal how changes in the independent variables between successive years are associated with changes in the dependent variables when following the same municipalities over time.

As the levels of the two dependent variables (sickness absence rate, average 12% and disability retirement rates (1%) were very different, these outcomes were used as logged dependent variables in the analyses in order to enable comparability of the results concerning the two outcomes. Thus, the results indicate the relative change in these rates when the explanatory variable changes by one unit [32].

First, the effect of change in the level of unemployment was examined on either change in the municipalities’ sickness absence rates or change in the municipalities’ disability retirement rates (model 1; the two dependent variables were examined in different sets of models). Covariates were added to the base model as blocks: first, adding variables on population, demographic and socioeconomic structures and morbidity (model 2), and second, adding variables on the municipalities’ economic situation (model 3). In addition, a model similar to the adjusted model 3 was run using only data for years 2007–2017 (model 4), for which years it was possible to also include variables on the industry structure (model 5). Correlation of residuals at the municipality level across different years was taken into account through robust standard errors when calculating the statistical significance on the estimates. The analyses were performed with R version 3.5.3 [33] utilizing the plm and lmtest packages [34,35].

## 3. Results

**Figure A2:**
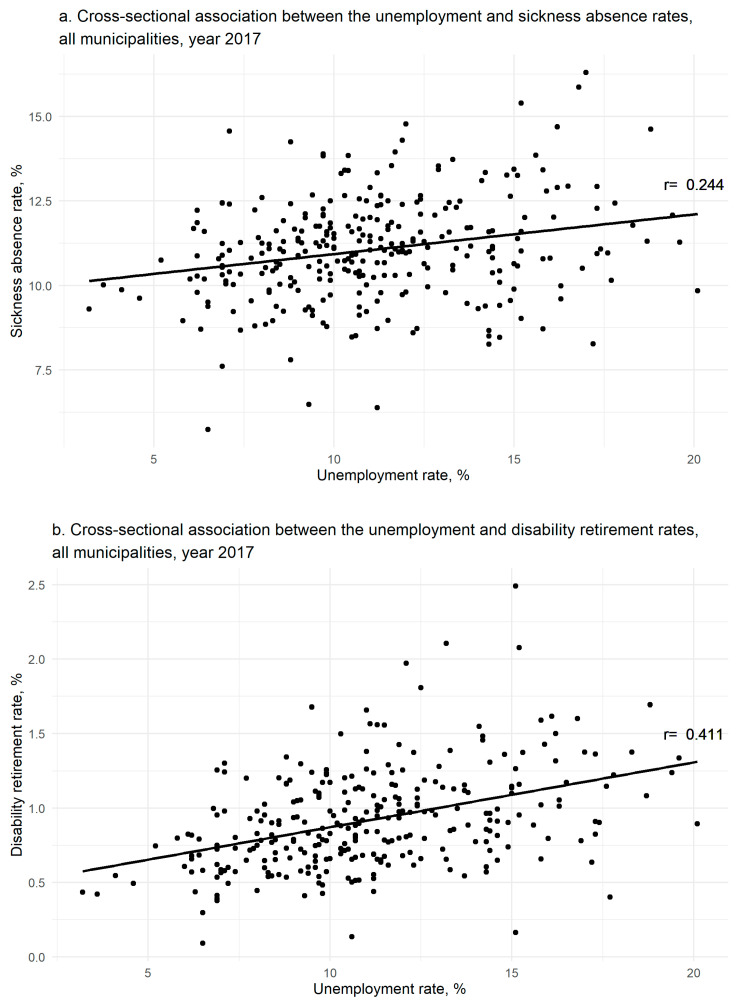
Cross-sectional associations of municipality-level unemployment rate with (**a**) sickness absence rate and (**b**) disability retirement rate in 2017.

To illustrate the between-municipality cross-sectional associations, Table 2 shows the cross-sectional yearly correlations between municipality-level unemployment rates and sickness absence or disability retirement rates. The correlation coefficients mostly show positive and statistically significant—albeit rather low—cross-sectional correlations, meaning that a higher unemployment rate of the municipality has generally been associated with both a higher sickness absence rate and a higher disability retirement rate. Figure A2 (Appendix A) visualises these cross-sectional associations with scatter plots for year 2017. Before year 2007, the correlations between unemployment and sickness absence rates were low and not statistically significant.

However, longitudinal analyses of within-municipality changes showed clear negative associations: the higher the unemployment rate, the lower both the sickness absence rate and the disability retirement rate. Table 3 and Table 4 show the results of fixed effects panel regression models with the municipality-level yearly sickness absence rate (Table 3) and yearly disability retirement rate (Table 4) as the outcomes. The results on unemployment are interpreted from the first row of the table, which also shows the % change (% ∆) in the dependent variables related to one percentage point change in the unemployment rate, in different models.

A one percentage point increase in the unemployment rate was associated with a statistically significant decrease in the sickness absence rate (−2.2%, *p* < 0.001) in the unadjusted model (Table 3, Model 1). Adjustments for simultaneous changes in population, demographic structure, socio-economic structure and morbidity (Model 2) resulted in some attenuation of the estimate (−1.7%). Some further attenuation of the estimate (−1.3%) was observed when variables on the economic situation of the municipalities were entered (Model 3). Both Model 4, with Model 3 variables for years 2007–2017, and Model 5, with added variables of industry structure, did not alter the result concerning unemployment. The estimate for unemployment was statistically highly significant in all models. In addition, changes in many of the covariates showed statistically significant associations with changes in the sickness absence rate in Models 4 and 5. In particular, changes in variables depicting population size, population density, educational level, tax revenue and proportion of employees working in manufacturing showed highly significant associations (*p* < 0.001) with changes in the sickness absence rate.

Regarding the disability retirement rate, a one percentage point increase in the unemployment rate was associated with a statistically significant decrease in the disability retirement rate (−3.0%, *p* < 0.001) (Table 4, Model 1). Again, including variables describing the population, demographic and socioeconomic structure and morbidity decreased the estimate (−1.9%, Model 2). No substantial change in the estimate (−2.0%) was observed when adding variables of the municipalities’ economic situation (Model 3). For the shorter observation period of years 2007–2017, the estimate for the latter model was the same, and inclusion of the variables of the self-sufficiency of jobs and industry structure had only a small effect (estimate in the final model: −2.1%, *p* = 0.011). Changes in the covariates depicting the educational level and general morbidity were also statistically highly significantly (*p* < 0.001) associated with changes in the disability retirement rate in Models 4 and 5. Changes in the population density and tax revenue were also associated with changes in disability retirement.

## 4. Discussion

### 4.1. Main Results and Comparison to Previous Studies

A large number of studies have demonstrated a positive individual-level association of unemployment with sickness absence and disability retirement [2,3,4,5,6,7,8,9,10,11]. However, many macro-level studies—especially those looking at trends in unemployment and sickness absence—have shown that sickness absence rates tend to decrease when unemployment increases, and vice versa [19,20,21,22,23]. To deepen the understanding of the interplay and dynamics between unemployment and disability benefit recipiency, we used fixed-effects models on time-series data concerning Finnish municipalities over 15 years to examine how changes in unemployment rates affect changes in sickness absence and disability retirement rates at the area level. We are not aware of previous studies examining these trends with longitudinal municipality-level or small area level data, even though positive cross-sectional correlations between unemployment and disability measures have been observed also using area-level data [16,17,18].

In our data, we also found that the unemployment rate was cross-sectionally positively correlated with both sickness absence and disability retirement rates at the municipality level: the higher the level of unemployment, the higher the level of sickness absence and disability retirement. Even though the correlation coefficients were rather modest, they were statistically highly significant during 2007–2017 concerning sickness absence rates and during 2003–2017 concerning disability retirement rates. However, the longitudinal analyses revealed a pro-cyclical pattern between unemployment and sickness absence and disability retirement rates. During the period 2003–2017, adjusted for simultaneous changes in a set of municipality-level covariates, a one percentage point increase in the unemployment rate was associated with a 1.3% decrease in the municipality-level sickness absence rate, i.e., in the proportion of working-age population on long-term sickness absence during the year. Likewise, a one percentage point increase in the unemployment rate was associated with a 2.1% decrease in the disability retirement rate, i.e., in the proportion of working-age population entering disability retirement during the year. The result can be interpreted also in the other direction: when unemployment decreases, take-up rates of disability benefits increase.

We adjusted for simultaneous temporal changes in several area-level covariates, such as demographic and socioeconomic structures, general morbidity and the economic level of the municipalities. These are factors that may play a role in the relationship between unemployment and disability benefit rates [11,16,25]. The association of unemployment rate with sickness absence and disability retirement rates was only partly attenuated after inclusion of the covariates in the models. Some interesting associations were found also concerning the covariates that were adjusted for, with the aim of tackling confounding associations. For example, an increase in the municipalities’ tax revenue per capita was associated with a decrease in the sickness absence rate but an increase in the disability retirement rate, net of changes in unemployment and other covariates. On the other hand, an increase in the educational level, in terms of the proportion of persons with a tertiary education, was associated with a decrease in both the sickness absence and the disability retirement rates. Further examination and explanation of these associations warrant future studies focusing on these indicators.

Our results concerning the pro-cyclical nature of sickness absence at the municipality level are in agreement with previous studies that have shown a similar relationship with nation-level data [20,21,22,23]. However, our results concerning the pro-cyclical nature of also disability retirement are in contrast to those obtained previously at the macro level from Iceland [29]. While the Icelandic study showed that increasing unemployment resulted in increasing disability retirement rolls, our study showed a negative association between the two measures. The diverging results may partly be explained by different settings, time frames, research designs and data sets used. The authors of the Icelandic study state that the level of the disability pension benefit is higher than the level of the universal unemployment benefit in Iceland. Thus, there has been a clear financial incentive for those becoming unemployed in Iceland to seek a disability pension even though remaining on unemployment benefits would be the more relevant option [29]. Those who become unemployed and suffer from work ability problems may therefore often transfer to disability retirement in Iceland, while in other countries, such as in Finland, they often continue to draw unemployment benefits.

Our results also showed that disability retirement may be even more pro-cyclical than sickness absence, as we found a slightly stronger association of unemployment with the disability retirement rate than with the sickness absence rate. An explanation for this stronger association with the disability retirement rate is that also at the individual level, unemployment seems to be more strongly linked to later disability retirement than to sickness absence [4]. Further, statistics show that among those who enter disability retirement, there is a larger share of persons with an unemployment background than among those having sickness absence spells [36,37]. Future studies, utilizing more varied data sets and preferably linking individual-level data to area-level data, need to find further explanations for this observation.

The benefit schemes for unemployment and disability are designed to tackle two intrinsically different types of risks in an individual’s life. However, it can be argued that unemployment and disability benefit systems are deeply intertwined and may in practice partly work as substitutes for each other. In some cases, they may be alternative benefit options for persons with a precarious labour market situation, often characterized by past periods of unemployment and by weaker than average health [18,38,39]. During economic downturns, those in most precarious situations and having poorest health are the first ones at risk of unemployment. This may then decrease the observed sickness absence rate and flow into disability retirement in the work force. Thus, when unemployment is higher, there is less pressure on the disability benefit systems since more persons with health problems are covered by the unemployment scheme and do not necessarily seek disability benefits as their subsistence is already settled for the time being. On the other hand, when the economy recovers again towards a better employment situation, unemployed persons with work ability problems may also move from unemployment to employment—but since their health is likely to be more fragile, they may often end up on sickness absence and thus increase the average sickness absence rate during economic booms. In addition, when employment prospects are higher during low levels of unemployment, their weak work ability may become visible to the point that they are admitted a disability pension. Beatty et al. [18,39] call this intertwining of unemployment and sickness/disability as “hidden unemployment” and “hidden sickness”, depending on which benefit seems to prevail in the ongoing economic cycle and benefit system.

Furthermore, the result of the positive area-level cross-sectional association despite the negative longitudinal association is understandable in light of the fact that there are areas that suffer simultaneously from both unemployment and disability problems. This may happen if there are few employment opportunities and a large share of individuals suffering from health-related or skills-related employability problems. Often these are remote areas characterized by economic decline and rather scarce employment possibilities, especially for those with weakened work ability [16,18,39]. In this respect, British studies have highlighted areas that have most suffered from a decline in industrial employment that has concerned especially low-skilled, older workers previously employed in traditional industries. In Finland, areas with both high unemployment and high disability benefit rates are often situated in the rather remote, sparsely populated regions in the Northern and Eastern parts of the country, which have also suffered from job loss, decreasing opportunities and population decline and where morbidity and disability is higher than in the Southern and Western part of the country [40,41].

Similarly, at the other end of the continuum, there are areas with lower than average levels of both unemployment and disability. In all types of areas, however, unemployment and disability rates may be equally intertwined. Thus, unemployment and disability measures taken together may thus be better indicators of a poor regional labour market situation and also of poor employability of the population than any of the variables alone.

In sum, the results demonstrate the tight connections between unemployment and disability that are visible at the area level. These phenomena probably affect partly the same people, who move between unemployment and disability benefit systems in time, depending on economic cycles. Our results also indicate that if the relationship between these phenomena is not understood, a flawed picture may prevail of either the prevalence of unemployment and/or disability in a given area. Furthermore, it has to be understood that a decrease in the unemployment rate is not only good news but may actually be associated with negative consequences in terms of increasing disability rates.

### 4.2. Strengths and Limitations

We were able to construct a rich, long panel of municipality-level data from reliable national databases, comprising cross-sectional time-series data on 295 municipalities over 15 years. Almost all variables were available for the total panel and thus there was very little missing information. However, relevant municipality-level variables may be missing from the analysis since some potentially important covariates could not be found in municipality-level databases or statistics. For example, we were not able to use operable variables on the service structure—either depicting the availability and quality of overall health care and social services, or more specifically variables on work ability services for the unemployed. Thus, there may still be some time-dependent confounding that we have not been able to take into account.

This study showed the importance of using panel data models also at the area level, when longitudinal data is available, to be able to trace the mechanisms working behind cross-sectional associations. Cross-sectional observations alone may yield one-sided and biased conclusions on the associations between unemployment and disability rates at the area level.

Due to differences in benefit systems, the results of this study may be generalized only with caution to other countries. However, as our results were largely in accordance with previous macro-level studies, the area-level temporal associations are likely to hold in many other contexts. Future research should study the associations and interplay between unemployment and sickness absence and disability retirement benefits in different social security systems with different types of benefits that tackle risks in life, thus taking into account different contexts, economic cycles and different conditions for receiving benefits. Further, the current unprecedented situation caused by the COVID-19 pandemic, which has rapidly increased both unemployment and morbidity and most likely has long-term consequences also on work ability, warrants future research on the associations between these phenomena.

## 5. Conclusions

This study showed that increasing unemployment is associated with decreasing sickness absence and disability retirement rates and, vice versa, decreasing unemployment is associated with increasing disability rates at the municipality level. Thus, booming economy and decreasing unemployment rates may bring about also unintended negative consequences such as increasing pressure and expenditure on the disability benefit schemes. On the other hand, it should be noted that decreases in the sickness absence and disability retirement rates may coincide with increasing unemployment rates. The observed temporal association may be explained with the correlation of the risks of disability and unemployment at the individual level. Work ability of those in precarious labour market positions should be monitored and their health problems tackled in the early stages. Unemployment and disability rates should more often be assessed together both at the national level and at the area level in order to reach a more complete understanding of the situation of those outside employment and of those suffering from work ability problems.

## Figures and Tables

**Table 1:** Descriptive statistics for the municipality-level variables, measured yearly in 2003–2017. All municipalities (*n* = 295) and years (*n* = 15) pooled, total *n* = 4425 observations.

Variable	Mean	Standard Deviation	Min	Max
Sickness absence rate: proportion (%) receiving sickness allowance of the non-retired population aged 18–64	12.0	1.7	5.3	18.3
Disability retirement rate: proportion (%) transferring to disability retirement of the non-retired population aged 18–64	1.0	0.4	0.0	3.1
Unemployment rate: proportion (%) unemployed of the labour force	11.0	3.8	2.3	26.8
Population size ^1^	18,119	45,690	734	643,272
Population density: population/km^2 1^	58	226	0.2	3051
Age structure: proportion (%) of persons aged 50 and over among population aged 18–64	40.4	6.3	23.1	58.5
Gender structure: proportion (%) of men among population aged 18–64	52.5	1.7	47.8	59.6
Immigrants: proportion (%) of population having immigrant background	2.0	1.8	0.1	18.0
Socio-economic structure: proportion (%) having tertiary education of population aged 15 and over	20.4	6.4	8.1	57.6
Poverty: proportion (%) of social assistance recipients of population aged 25–64	6.2	2.2	1.1	15.7
Morbidity: general age-and gender-adjusted morbidity index (100 = country total in each year)	108.7	15.2	62.6	163.6
Inactive/active ratio: number of those not employed relative to 100 employed persons	159.4	31.1	91.8	265.0
Municipal economy: tax revenue, € per capita, in 2017 money ^2^	3128	608	1661	7198
Self-sufficiency in jobs: number of jobs at workplaces in the municipality relative to 100 employed persons	86.7	17.7	39.0	149.9
Industry structure 1: proportion (%) of population of the labour force working in the manufacturing sector ^3^	16.1	7.0	0.0	53.6
Industry structure 2: proportion (%) of population of the labour force working in the construction sector ^3^	7.6	2.0	1.1	20.7
Industry structure 3: proportion (%) of population working in health and social services, education, social insurance and public administration ^3^	27.8	4.2	11.1	53.3

^1^ Used as a logged variable in the analyses. ^2^ Transformed to the scale of 100 euros in subsequent models. ^3^ Available for 2007–2017.

**Table 2:** Cross-sectional yearly correlations of municipality-level unemployment rates with sickness absence and disability retirement rates in 2003–2017.

Year	Correlation between Unemployment and Sickness Absence Rates		Correlation between Unemployment and Disability Retirement Rates	
	Pearson’s R	Significance ^1^	Pearson’s R	Significance ^1^
2003	−0.015	Ns.	0.336	***
2004	0.102	Ns.	0.388	***
2005	0.114	Ns.	0.375	***
2006	0.068	Ns.	0.455	***
2007	0.196	***	0.422	***
2008	0.208	***	0.461	***
2009	0.274	***	0.471	***
2010	0.304	***	0.433	***
2011	0.207	***	0.394	***
2012	0.217	***	0.385	***
2013	0.243	***	0.393	***
2014	0.265	***	0.347	***
2015	0.230	***	0.351	***
2016	0.259	***	0.373	***
2017	0.244	***	0.411	***

^1^ Ns. = Not statistically significant. *** = *p* < 0.001.

**Table 3:** Results on sickness absence (logged sickness absence rate as outcome). Fixed effects models on Finnish municipalities (*n* = 295) over 15 years (2003–2017).

Independent Variable	Model 1 ^1^			Model 2			Model 3			Model 4			Model 5		
	B	% ∆ ^2^	*p*	B	% ∆	*p*	B	% ∆	*p*	B	% ∆	*p*	B	% ∆	*p*
Unemployment rate	−0.02	−2.2	<0.001	−0.02	−1.7	<0.001	−0.01	−1.3	<0.001	−0.01	−1.3	<0.001	−0.01	−1.3	<0.001
Log population				−0.24	−0.2	0.164	−0.28	−0.3	0.108	−0.48	−0.5	<0.001	−0.44	−0.4	<0.001
Log population density				0.23	0.2	0.176	0.24	0.2	0.153	0.39	0.4	<0.001	0.38	0.4	<0.001
% age 50+				0.00	0.0	0.777	0.00	0.1	0.377	0.00	0.2	0.226	0.00	0.2	0.230
% men				−0.01	−0.8	0.098	−0.01	−0.8	0.087	−0.01	−1.2	0.015	−0.01	−1.1	0.020
% immigrant background				−0.01	−1.2	<0.001	−0.01	−1.0	<0.001	−0.01	−1.0	0.002	−0.01	−0.9	0.002
% tertiary education				−0.02	−2.2	<0.001	−0.02	−1.5	<0.001	−0.02	−1.9	<0.001	−0.02	−1.8	<0.001
% social assistance recipients				0.00	−0.4	0.064	0.00	−0.3	0.113	0.00	−0.4	0.050	0.00	−0.4	0.048
Morbidity index				0.00	0.0	0.351	0.00	0.1	0.134	0.00	0.1	0.158	0.00	0.1	0.136
Inactive/active ratio							0.00	−0.1	<0.001	0.00	0.0	0.148	0.00	0.0	0.394
Tax revenue, 100 € per capita							0.00	−0.4	<0.001	0.00	−0.4	<0.001	0.00	−0.4	<0.001
Self-sufficiency in jobs							0.00	0.1	0.222	0.00	0.0	0.558	0.00	0.0	0.883
% working in manufacturing													0.00	0.4	<0.001
% working in construction													0.00	−0.2	0.246
% working in public administration and services													0.00	0.3	0.004
N of observations	4425			4425			4425			3245			3245		
Years in follow-up	2003–2017			2003–2017			2003–2017			2007–2017			2007–2017		

^1^ Each model includes those independent variables for which estimates that are shown in the Table. ^2^ % change in the dependent variable when the independent variables increases one unit. Concerning non-logged independent variables, % ∆ calculated as 100×[exp(β) − 1], and concerning logged independent variables (log population and log population density), % ∆ ≈ β [32].

**Table 4:** Results on disability retirement (logged disability retirement rate as outcome). Fixed effects models on Finnish municipalities (*n* = 295) over 15 years (2003–2017).

Independent Variable	Model 1 ^1^			Model 2			Model 3			Model 4			Model 5		
	B	% ∆ ^2^	*p*	B	% ∆	*p*	B	% ∆	*p*	B	% ∆	*p*	B	% ∆	*p*
Unemployment rate	−0.03	−3.0	<0.001	−0.02	−1.9	<0.001	−0.02	−2.0	0.003	−0.02	−2.0	0.027	−0.02	−2.1	0.011
Log population				0.18	0.2	0.673	0.21	0.2	0.623	−0.19	−0.2	0.708	−0.18	−0.2	0.714
Log population density				0.63	0.6	0.086	0.61	0.6	0.093	1.07	1.1	0.022	1.05	1.0	0.023
% age 50+				0.01	1.2	<0.001	0.01	1.1	0.001	0.00	0.0	0.985	0.00	−0.2	0.885
% men				−0.06	−5.9	0.362	−0.06	−5.9	0.367	−0.08	−7.3	0.391	−0.07	−7.1	0.391
% immigrant background				0.00	0.0	0.993	0.00	−0.2	0.917	−0.01	−0.6	0.783	−0.01	−0.6	0.811
% tertiary education				−0.07	−6.4	<0.001	−0.07	−7.0	<0.001	−0.08	−8.1	<0.001	−0.09	−8.3	<0.001
% social assistance recipients				0.00	0.1	0.897	0.00	0.1	0.890	−0.01	−0.6	0.563	0.00	−0.5	0.617
Morbidity index				0.02	1.6	<0.001	0.02	1.6	<0.001	0.02	1.9	<0.001	0.02	1.8	<0.001
Inactive/active ratio							0.00	0.1	0.756	0.00	0.1	0.555	0.00	0.1	0.714
Tax revenue, 100 € per capita							0.00	0.5	0.286	0.01	1.2	0.014	0.01	1.3	0.013
Self-sufficiency in jobs							0.00	−0.1	0.642	0.00	−0.2	0.263	0.00	−0.2	0.243
% working in manufacturing													0.00	−0.2	0.759
% working in construction													−0.01	−0.8	0.583
% working in public administration and services													0.00	0.5	0.345
N of observations	4425			4425			4425			3245			3245		
Years in follow-up	2003–2017			2003–2017			2003–2017			2007–2017			2007–2017		

^1^ Each model includes those independent variables for which estimates that are shown in the Table. ^2^ % change in the dependent variable when the independent variables increases one unit. Concerning non-logged independent variables, % ∆ calculated as 100[exp(β) − 1], and concerning logged independent variables (log population and log population density), % ∆ ≈ β [32].

## Data Availability

Data are available from statistical databases mentioned in the article and also available from the corresponding author by request.

## Appendix A

**Table A1:** Sources of the municipality-level variables.

Variable	Variable Source
Sickness absence rate: proportion (%) receiving sickness allowance of the non-retired population aged 18–64	Calculated using unpublished statistics from Kela ^1^ and population data from the StatFin database of Statistics Finland
Disability retirement rate: proportion (%) transferring to disability retirement of the non-retired population aged 18–64	Calculated using unpublished statistics of Finnish Centre for Pensions and population data from the StatFin database of Statistics Finland
Unemployment rate: proportion (%) unemployed of the labour force	Sotkanet database of THL ^2^
Population size	Sotkanet database of THL
Population density: population/km^2^	StatFin database of Statistics Finland
Age structure: proportion (%) of persons aged 50 and over among population aged 18–64	Calculated from population frequency data from the database of Kela
Gender structure: proportion (%) of men among population aged 18–64	Calculated from population frequency data from the database of Kela
Immigrants: proportion (%) of population having immigrant background	StatFin database of Statistics Finland
Socio-economic structure: proportion (%) having tertiary education of population aged 15 and over	Sotkanet database of THL
Poverty: proportion (%) of social assistance recipients of population aged 25–64	Sotkanet database of THL
Morbidity: general age-and gender-adjusted morbidity index (100 = country total in each year)	Sotkanet database of THL
Inactive/active ratio: number of those not employed relative to 100 employed persons	StatFin database of Statistics Finland
Municipal economy: tax revenue, € per capita, in 2017 money	Sotkanet database, inflation corrected
Self-sufficiency in jobs: number of jobs at workplaces in the municipality relative to 100 employed persons	Municipality indicators database of Statistics Finland
Industry structure 1: proportion (%) of population of the labour force working in the manufacturing sector	Sotkanet database of THL
Industry structure 2: proportion (%) of population of the labour force working in the construction sector	Sotkanet database of THL
Industry structure 3: proportion (%) of population working in health and social services, education, social insurance and public administration	Sotkanet database of THL

^1^ Kela = the Social Insurance Institution of Finland. ^2^ THL = Finnish National Institute for Health and Welfare.
